# Risk factors for colonization with multidrug-resistant Gram-negative bacteria and *Clostridioides difficile* in Long Term Care Facilities (LTCFs) residents: the evidence from 27 facilities in a high endemic setting

**DOI:** 10.3389/fcimb.2023.1155320

**Published:** 2023-06-12

**Authors:** Anna Maria Azzini, Giorgia Be, Laura Naso, Lorenza Lambertenghi, Nicola Duccio Salerno, Ilaria Coledan, Alda Bazaj, Massimo Mirandola, Jessica Miotti, Fulvia Mazzaferri, Simone Accordini, Giuliana Lo Cascio, Evelina Tacconelli

**Affiliations:** ^1^ Infectious Diseases Division, Department of Diagnostics and Public Health, University of Verona, Verona, Italy; ^2^ Infectious Disease Unit, Mater Salutis Hospital - ULSS 9 Scaligera, Legnago, Italy; ^3^ Microbiology and Virology Unit, AOUI Verona, Verona, Italy; ^4^ SerT Suzzara, SC Ser.D. Mantova, ASST Mantova, Suzzara, Italy; ^5^ Microbiology Division, Department of Diagnostics and Public Health, University of Verona, Verona, Italy; ^6^ Unit of Epidemiology and Medical Statistics, Department of Diagnostics and Public Health, University of Verona, Verona, Italy; ^7^ Microbiology Unit, AUSL Piacenza, Piacenza, Italy

**Keywords:** LTCF, colonization, ESBL, carbapenem-resistant, carbapenemase, *C. difficile*, risk factors, epidemiology

## Abstract

**Introduction:**

Residency in LTCFs increases the likelihood of colonization with multidrug resistant Gram-negative bacteria (MDR-GNB). We assessed the prevalence and risk factors for enteric colonization by III-generation cephalosporins-resistant and carbapenem-resistant (CR) GNB in a large group of LTCFs in a high endemic setting. We also assessed the prevalence and risk factors for *C. difficile* colonization.

**Methods:**

A point prevalence survey with rectal screening (RS) was conducted in 27 LTCFs in north Italy. Epidemiological and clinical variables on the survey day, history of hospitalization and surgery within one year, and antibiotics within three months, were collected. The presence of III-generation cephalosporin resistant and CR GNB was assessed using a selective culture on chromogenic medium and PCR for carbapenemase detection. The presence of *C. difficile* was assessed using ELISA for GDH and RT-PCR to identify toxigenic strains. Multi-variable analyses were performed using two-level logistic regression models.

**Results:**

In the study period 1947 RSs were performed. The prevalence of colonization by at least one GNB resistant to III-generation cephalosporin was 51% (*E. coli* 65%, *K. pneumoniae* 14% of isolates). The prevalence of colonization by CR GNB was 6%. 6% of all isolates (1150 strains) resulted in a carbapenem-resistant *K. pneumoniae*, and 3% in a carbapenem-resistant *E. coli*. KPC was the most frequent carbapenemase (73%) identified by PCR, followed by VIM (23%). The prevalence of colonization by *C. difficile* was 11.7%. The presence of a medical device (OR 2.67) and previous antibiotic use (OR 1.48) were significantly associated with III-generation cephalosporin resistant GNB colonization. The presence of a medical device (OR 2.67) and previous hospitalization (OR 1.80) were significantly associated with CR GNB. The presence of a medical device (OR 2.30) was significantly associated with *C. difficile* colonization. Main previously used antibiotic classes were fluoroquinolones (32% of previously treated subjects), III-generation cephalosporins (21%), and penicillins (19%).

**Conclusion:**

Antimicrobial stewardship in LTCFs is a critical issue, being previous antibiotic treatment a risk factor for colonization by MDR-GNB. The prevalence of colonization by III-generation cephalosporin and CR GNB among LTCF residents also underlines the importance to adhere to hand hygiene indications, infection prevention and control measures, and environmental hygiene protocols, more achievable than rigorous contact precautions in this type of social setting.

## 1 Introduction

The increase in the elderly population in a social context characterized by the ever-decreasing availability and possibility of support and care within the family nucleus, have determined the strengthening of assistance in *ad hoc* long-term care facilities (LTCFs), such as nursing homes, residential care centers, chronic disease hospitals, and rehabilitation centers (Strausbaugh et al., 1996; OECD, 2011; Suetens, 2012). Although the numbers vary from country to country, approximately 2–5% of the developed world’s older population resides in some type of LTCFs (Ribbe et al., 1997).

The elderly has an increased risk of developing infections (van Duin, 2012), due both to physiological changes linked to aging (Castle, 2000; Heppner et al., 2013), to the onset of chronic diseases (Juthani-Mehta and Quagliarello, 2010; Falcone et al., 2021), and to the need for medical devices (e.g., urinary catheter) (Wang et al., 2012). Infections are the most common cause of LTCFs’ residents morbidity and mortality and transfers to acute care hospitals (Burns et al., 2015; Childs et al., 2019), therefore, it is not surprising that antibiotics are among the most prescribed medications in this setting (Nicolle et al., 2000; Latour et al., 2012). According to point prevalence surveys (PPSs) in Europe, USA, and Australia, between 3% and 15% of LTCF residents are given antibiotics at any time (McClean et al., 2012; Stuart et al., 2012; van Buul et al., 2012), and there is a 40-70% likelihood of exposure to at least one course of antibiotics for residents who remain in a nursing home for at least 6 months (Benoit et al., 2008). Around half of these treatments are inappropriate or unnecessary thus increasing the potential for the selection of multi-drug resistant bacteria (Loeb, 2000; Nicolle et al., 2000; Gavazzi and Krause, 2002; McClean et al., 2011; Rotjanapan et al., 2011; Drinka et al., 2013). In 2013 the PPS on healthcare associated infections and antimicrobial use in European LTCFs (HALT 2) documented that 4.4% of the subjects admitted to the involved LTCFs were taking at least one antibiotic, but in half of the cases in the absence of a healthcare-related infection (ECDC-WHO). Similar results were described by HALT 3 in 2017 (Ricchizzi et al., 2018), with common causes of antibiotic misuse in LTCFs the unnecessary antibiotic treatments for bacterial colonization (e.g., asymptomatic bacteriuria), for urinary tract infection prophylaxis, or for viral infections (e.g., influenza), and the longer-than-necessary antibiotic’s course duration in the absence of treatment reassessment at around day 3 after its prescription (Dyar et al., 2015).

Prior antibiotic treatments, as well as the total dependence of residents on nurse care for their daily living activities, expose them both to the selection and horizontal transmission of antibiotic resistant organisms, in a generally understaffed residential setting where is difficult to strictly adhere to infection prevention and control strategies and to hand hygiene indications (Arena et al., 2018; Ambretti et al., 2019).

The emergence of multidrug-resistant organisms (MDROs) is a major public health concern (Infectious Diseases Society of America (IDSA) et al., 2011). In 2020 in Italy, as in other south-eastern EU-EEA countries, more than 25% of *E. coli* and more than 50% of *K. pneumoniae* invasive isolates were resistant to III-generation cephalosporins, percentages of carbapenem resistance above 10% were reported in *P. aeruginosa* and even higher in *K. pneumoniae* (25%-50%) and in *Acinetobacter* spp. (> 50%), thus describing a highly endemic setting (European Centre for Disease Prevention and Control, 2022).

Multi-drug antibiotic resistance also affects residents in LTCFs that, infected or colonized, become a reservoir (Willemsen et al., 2015; Ruiz-Garbajosa et al., 2016; van den Dool et al., 2016; Nucleo et al., 2018; Nguyen et al., 2019): extended-spectrum ß-lactamase (ESBL) *Enterobacterales*, carbapenem-resistant *Enterobacterales*, multidrug resistant *P. aeruginosa* and *A. baumannii* are increasing in prevalence inside LTCFs (Perez et al., 2007; Willemsen et al., 2015; Ruiz-Garbajosa et al., 2016; van Duin and Paterson, 2016; Nucleo et al., 2018; Horcajada et al., 2019). Although it is not an MDRO, also *Clostridioides difficile* represents a worldwide public health concern, as it is one of the major causes of antibiotic-associated infections in healthcare settings, especially among older people living in nursing homes (Barbut et al., 2011; Rodriguez et al., 2014).

### 1.1 Aims of the study

Knowledge of individual level risk factors for colonization in LTCFs’ residents is mainly limited to small groups. A recent systematic review identified studies with a small sample size and LTCFs across countries differing in case-mix, age and care provided (Flokas et al., 2017), hampering the possibility of defining the exact burden of the problem. The aim of the present study was to describe the prevalence and risk factors for enteric colonization by *C. difficile*, III-generation cephalosporin resistant gram-negative bacteria (GNB) and CR GNB in a large sample of residents of 27 LTCFs in Veneto, a Northern Italian region.

## 2 Materials and methods

### 2.1 Setting

Between July 2018 and June 2019, we conducted a PPS to assess both the frequency of healthcare-related infections and the enteric colonization status by MDR Gram negative bacteria and *C. difficile* in the population of elderly residents of 27 LTCFs in the Veneto Region. Participation in the survey was voluntary, but at least one facility for each province of the region was enrolled. The 27 facilities were not involved simultaneously but at different times based on the local Ethic Committee’s approval, the willingness of local personnel to collaborate with researchers in the collection of the study-specific biological samples and the possibility of the reference microbiology laboratory to accept and process the samples. The survey was proposed to all patients living full-time in the LTCF, present in the facility on the day of the PPS and admitted at least 48 hours before the survey. Only patients who were able to provide written informed consent, or whose legal representative consented, were enrolled. The survey was carried out in a single day for each facility. In the case of facilities with more than 50 residents living in different wards, the survey lasted several consecutive days, however, all the beds in one ward had to be surveyed on the same day. For each enrolled patient, the variables measured included the type and etiology of concurrent infections and respective antibiotic therapy, any antibiotic treatments in the previous 3 months, hospital admissions and surgery in the previous 12 months and case mix factors, such as presence of urinary catheter, vascular catheter, pressure sores, skin wounds other than bedsores, urinary and/or fecal incontinence, low mental status, and impaired mobility. For each enrolled host, a rectal swab was performed to assess the status of colonization by MDR Gram negative bacteria (*Enterobacterales* and non-fermenting GNB resistant to III-generation cephalosporin and/or carbapenem). On the PPS day, from each enrolled resident and independently from the presence of acute gastrointestinal symptoms, a fecal sample was also taken in sterile collection cups, refrigerated and transported to the laboratory within 24 hours to assess the *C.difficile* colonization status.

### 2.2 Strains detection

For screening of III-generation cephalosporin and carbapenem-resistant GNB, rectal swabs were inoculated onto ChromID ESBL agar (bioMerieux, Marcy l’Etoile, France) with an Ertapenem disk (10ug) and on Mac Conkey agar with a Meropenem disk (10ug) (Carrër et al., 2010). Plates were incubated at 35 ± 2°C under aerobic conditions for 24h. Isolates were identified at the species level using the MALDI-TOF Vitek MS System (bioMerieux, Marcy l’Etoile, France). Resistance to carbapenems were interpreted according EUCAST criteria and Italian AMCLI (Associazione Microbiologi Clinici Italiani) recommendations (Eucast; Aschbacher et al., 2020). A diameter zone < 30 mm for meropenem on MacConkey and/or < 28 mm for ertapenem on ChromID ESBL agar were considered suspicious for carbapenem resistance and the colony growth within this size zone was confirmed with immunochromatographic lateral flow assay NG-test Carba5 (NG Biotech) (from now Carba5 test). The Vitek2 system (bioMerieux, Marcy l’Etoile, France) was used to perform antimicrobial susceptibilities and to confirm carbapenem resistance in the selected strains (Hrabák et al., 2014).

All carbapenem-resistant strains detected by screening culture and confirmed by Carba5 test were further investigated by a home-made PCR using published primers and conditions (Poirel et al., 2011), targeting *bla*_KPC,_ *bla*_VIM_, *bla*_IMP,_ *bla*_OXA-48_, *bla*_NDM,_ *bla*_OXA-23_ and *bla*_OXA-24_ type genes, to identify on single isolated strains the presence of specific genes encoding for carbapenemase. All fecal samples were analyzed with Premier C. difficile GDH qualitative enzyme immunoassay (*Meridian* Bioscience Europe S.r.l.- Italy) screening test to detect *Clostridioides difficile* glutamate dehydrogenase (GDH) (Terveer et al., 2017; Leitner et al., 2020). All GDH-positive specimens were further tested with Illumigene® (*Meridian* Bioscience Europe S.r.l.- Italy), a loop-mediated isothermal amplification assay (LAMP) to detect a gene segment of the *tcdA* gene in the pathogenicity locus (PaLoc) present in all known toxigenic *C. difficile* strains.

### 2.3 Statistical analyses

Continuous variables were summarized as means with standard deviations (SD). Percentages were calculated for categorical variables. Two-level (level 1 unit: subject; level 2 unit: LTCF) random-intercept logistic regression models were used to assess the association of surgery in the previous 12 months, presence of at least one device, antibiotic treatment in the previous 3 months, and hospitalizations in the previous 12 months (potential risk factors) with rectal colonization by III-generation cephalosporins-resistant GNB, by carbapenem-resistant GNB, or by *C. difficile* (three different outcomes). Gender and age were included as adjustment variables in the three models. Tetrachoric or biserial correlation coefficients were computed to assess collinearity of two covariates. Surgery in the previous 12 months was dropped from the models because of collinearity with previous hospitalizations (tetrachoric correlation coefficient = 0.85) and because of data sparseness (only 3% of the subjects had undergone surgical procedures in the past year). All statistical analyses were performed using STATA software, version 17 (College Station, TX: StataCorp LP).

### 2.4 Ethics statement

This study was approved by the Ethics Committee for Clinical Trials of the Provinces of Verona and Rovigo: n° 25294, 26/04/2018.

## 3 Results

27 LTCFs were included in the survey. Based on the patient’s frailty degree and consequently on the intensity of requested daily assistance, half of these LTCFs (12 facilities) were classified as general nursing homes (providing principally care to seniors with severe illnesses or injuries), 8 as residential homes (usually providing personal care, housekeeping and three meals a day), 4 as mixed LTCF (providing mixed services for elderly or other resident populations, such as those who necessitate rehabilitation or palliative care) (Nucleo et al., 2018). For the remaining 3 facilities the typology of assistance was not provided. At the time of the PPS, the facilities had an average of 162 total beds (mean, SD ± 202), with an occupancy rate of 96.2%. All facilities offered continuous and permanent nursing care, with a mean of 27 nurses available 24h/24h (median 16.5), joined by a mean of 64 (median 45) auxiliary healthcare staff responsible mainly for carrying out the personal hygiene of the hosts. None of the facilities provided an internal medical doctor, but nearly all of them offered medical assistance through the support of external general practitioners associated to the facility by means of a formal agreement with the local public health authority.

From an overall population of 2983 subjects, 1947 residents were enrolled: 1440 women and 507 men (**Table 1**). The frequency of participation to the study fluctuated between 59% and 76% in 25 of the 27 structures involved; in a single facility with 150 residents, it reached 94%, while the lowest adhesion (38%) was obtained in a facility hosting 90 individuals. Participation in the study was mainly influenced by the difficulty in obtaining the informed consent from a population of subjects with cognitive impairment and/or without a legal representative. The mean age of the population was 84.97 years (range 38-115; SD ± 9.71), and more than half were older than 85 years. About 85% (1673/1947) of the subjects resided in the facility for at least 12 months, with an average LTCF-stay of 5.19 years (SD ± 14.77). 14% (282/1947) of residents had been hospitalized at least once during the previous twelve months: half of the hospitalizations occurring in a medical or geriatric setting and lasting an average of 17 days, and one fifth occurring in a specialized medical setting. 8% of residents had been hospitalized in a surgical setting, but only 60 subjects (3%) had undergone surgery in the previous year and 11 (0.5%) in the previous month. 25% (488/1947) of the enrolled patients were totally bedridden and 54% (1061/1947) showed mobility impairment; bedsores and trophic ulcers interested 346 patients (18%). Nearly two-thirds of the studied population presented fecal (1304/1947) and/or urinary (1328/1947) incontinence and more than 75% (1502/1947) had mental impairment from mild severity to stupor and confusion. On the day the survey was conducted, 382 residents (19%) had in place at least one medical device, and 118 of them had more than one. As described in **Table 2**, the most frequently used medical device was the urinary catheter, followed by endovascular catheter, and percutaneous endoscopic gastrostomy. There were no patients carrying abdominal drainage.

**Table 1:** Characteristics of the Long-Term Care Facilities’ enrolled patients.

Variable	Enrolled population N (%)
Enrolled residents, n (%)	1947 (100%)
Male, n (%)	507 (26%)
Age, years, mean (range; SD)	84.97 (range 38-115; SD ± 9.71)
Age > 85 years, n (%)	1192 (61%)
Surgical procedures in previous 12 months (major or minor surgery), n (%)	60 (3%)
Surgical procedures in previous 30 days (major or minor surgery), n (%)	11 (0.5%)
Hospital admissions in previous 12 months (any hospital setting), n (%)	282 (14%)
LTCF-stay > 1 year, n (%)	1673 (86%)
Bedridden, n (%)	488 (25%)
Mobility impairment, n (%)	1061(54%)
Mental impairment, n (%)	1502 (77%)
Fecal incontinence, n (%)	1304 (67%)
Urinary incontinence, n (%)	1328 (68%)
Bedsores, n (%)	253 (13%)
Trophic ulcers, n (%)	93 (5%)
Use of antiacids (proton pump inhibitors), n (%)	1280 (64%)

**Table 2:** Enrolled populations’ medical devices and underlying health impairment.

Variable	Enrolled population (N=1947)
At least one medical device (any type), n (%)	382 (19%)
Urinary catheter, n (%)	213 (11%)
Endovascular catheter (peripheral or central), n (%)	131 (7%)
PEG, n (%)	91 (5%)
Tracheostomy, n (%)	26 (1%)
Nasogastric tube, n (%)	9 (0.4%)
Others (urostomy, colostomy), n (%)	22 (1%)
Abdominal drainage, n (%)	0 (0%)
At least one disease, n (%)	1510 (77%)
Diabetes, n (%)	433 (22%)
COPD, n (%)	226 (11%)
Active cancer, n (%)	180 (9%)
Cirrhosis, n (%)	115 (6%)
IBD, n (%)	62 (3%)
Immunodeficiency *, n (%)	66 (3%)
End stage renal disease, n (%)	19 (1%)

PEG, percutaneous endoscopic gastrostomy; COPD, chronic obstructive pulmonary disease; IBD, inflammatory bowel disease; * both HIV chronic infection, cancer chemotherapy and steroid or other immunosuppressant treatment.

In our sample, 1510 patients (77%) presented at least one cause of health impairment (**Table 2**), diabetes was the most represented (22%, 433/1947), followed by COPD (11%, 226/1947) and active cancer (9%, 180/1947). Immunodeficiency secondary to active antineoplastic treatment or steroids or other immunosuppressant drugs interested 3% (57/1947) of the population. 9 subjects had chronic HIV infection.

On the PPS day, only 22 subjects (1%) were on antimicrobial therapy. All of them had at least one healthcare associated infection, for a total of 25 separate infective episodes (**Table 3**). Etiological diagnosis was rarely available; almost all the infectious episodes except one had been acquired within the facility.

**Table 3:** Healthcare associated infections (HAI).

HAI typology	Number of episodes N=25	Etiology
Pneumonia	13	-*P.aeruginosa* -*E.coli* -*K.pneumoniae*
Urinary tract infections	1	-*E.coli*
Skin and soft tissue infections	2	*/*
Sepsis	1	-*K.pneumoniae*
Other	8	/

### 3.1 Previous antibiotic treatment

567 subjects (29%) had taken at least one antibiotic in previous 90 days, 61% of which only one drug. The preferred route of administration had been the oral one (68%, 596/873 prescribed molecules), followed by the intravenous one (29%, 255/873). 76% of all 873 prescribed drugs had a therapeutic indication, mainly for respiratory tract infections (43%) and lower urinary tract infections (33%), but in 12% of them it was not possible to trace the actual indication. The remaining 179 prescriptions had a prophylactic indication, above all for the prevention of urinary tract infection in catheterized patient (25%) and of enteritis in patients with relapsing diarrhea (25%), but a clear indication was not available in about half of these prescriptions. Almost the totality of all prescriptions had been made inside the LTCFs (91%), while the rest had been prescribed in hospital, during previous hospitalization. **Table 4** describes all the antibiotics prescribed in previous 90 days for any indication.

**Table 4:** Antibiotics prescribed in previous 90 days, any indication.

Antimicrobial class	Treated patients (N 567)	Overall prescribed treatment (N 873)
Fluoroquinolones, n (%)	185 (32%)	213 (24.5%)
I generation cephalosporins, n (%)	2 (0.3%)	2 (0.25%)
III generation cephalosporins, n (%)	123 (21.7%)	109 (12%)
IV generation cephalosporins, n (%)	2 (0.3%)	2 (0.25%)
Penicillins, n (%)	108 (19%)	298 (34%)
Cotrimoxazole, n (%)	42 (7%)	43 (5%)
Nitrofurantoin, n (%)	16 (3%)	18 (2%)
Fosfomycin, n (%)	31 (5%)	33 (4%)
Glycopeptides, n (%)	4 (0.7%)	4 (0.5%)
Metronidazole, n (%)	4 (0.7%)	4 (0.5%)
Other, n (%)	50 (9%)	147 (17%)

### 3.2 Enteric colonization by multi drug-resistant Gram-negative bacteria

Overall, 1947 rectal swabs were collected, one for each enrolled patient. By selective culture, 991 (51%) subjects were colonized by at least one strain resistant to III-generation cephalosporins, in 15% of colonized subjects the rectal swab tested positive for two, and in 2% for three different strains. 119 patients (6% of the entire enlisted population) were colonized by at least one CR GNB.

**Figure 1:**
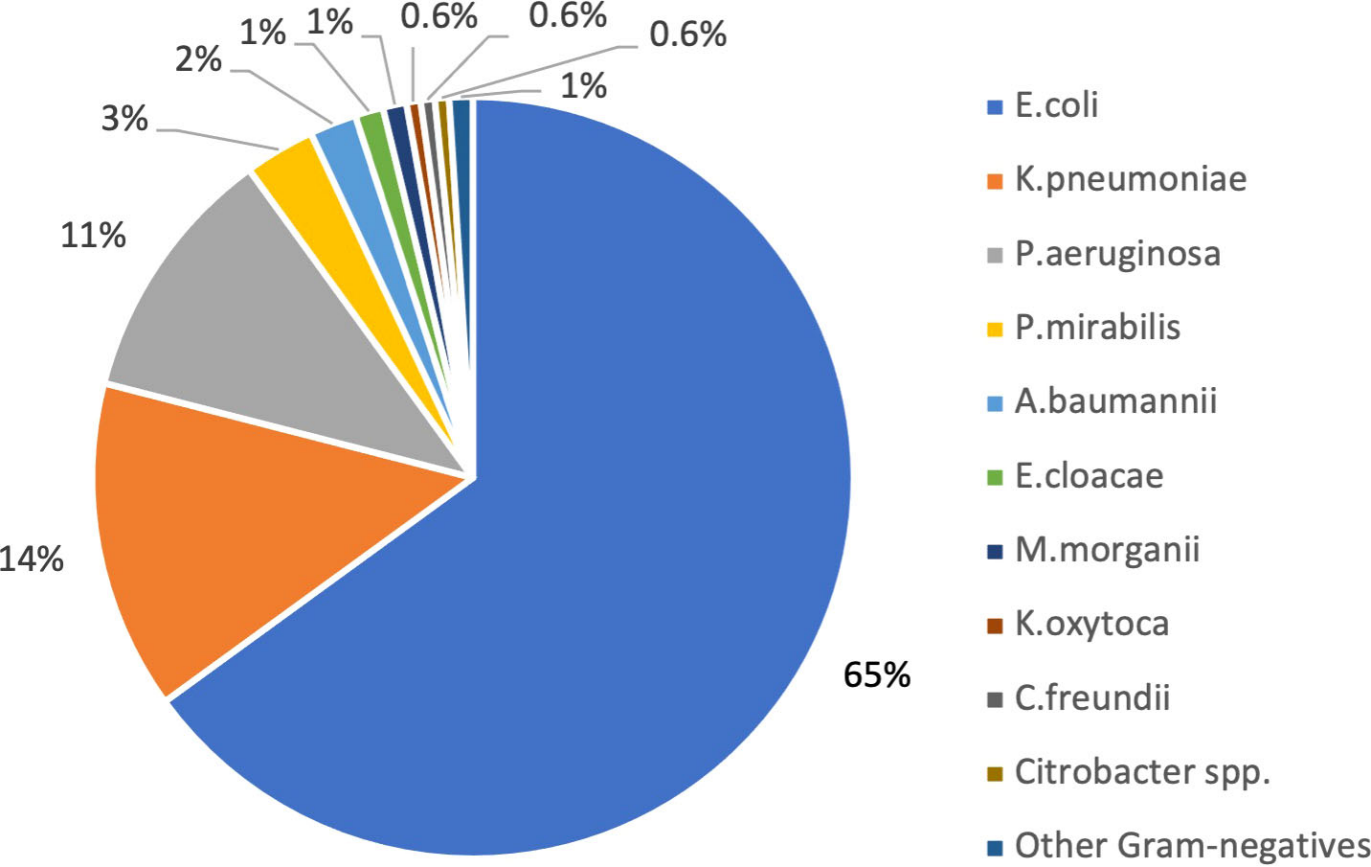
Gram negative bacteria isolated on rectal swab by selective ChromID ESBL agar medium (N 1150).

**Figure 2:**
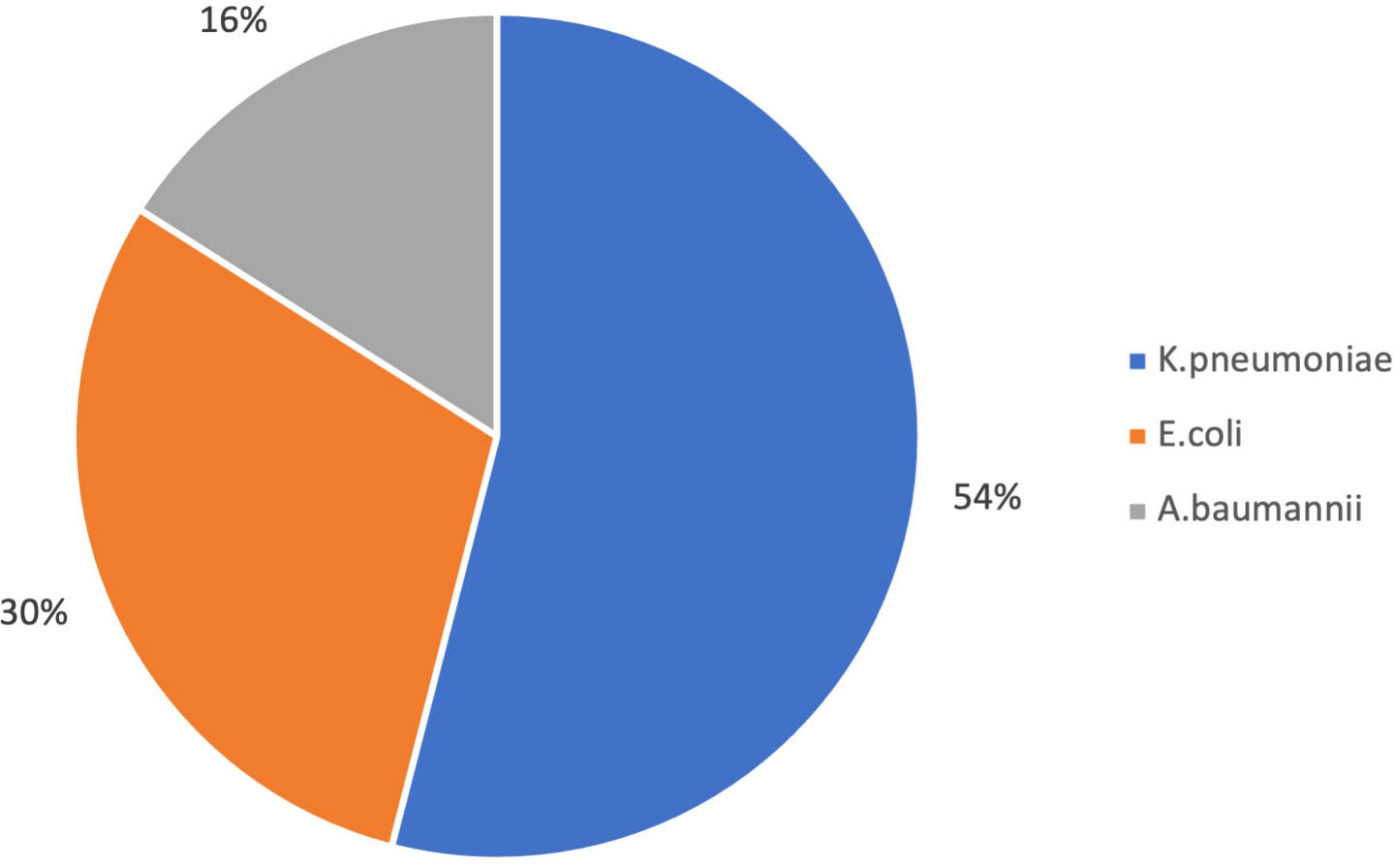
Carbapenem-resistant Gram negative bacteria isolated on rectal swab by selective culture (N 125).

Overall, 1150 gram-negative strains growing on ChromID ESBL agar were identified (**Figure 1**), 125 of which (11%) resulted carbapenem-resistant on ertapenem-meropenem disk diffusion test (**Figure 2**).

Carba5 test showed the presence of carbapenemases in 78 of them, corresponding to a prevalence of 3.5% patients colonized by at least one carbapenemase-producing micro-organisms. In particular Carba5 test detected a carbapenemase in 75% of carbapenem-resistant *Enterobacterales* and, as expected, in 0% of *A.baumannii* strains carbapenem-resistant on selective culture (**Table 5**).

**Table 5:** Comparison between NG-test Carba5 and home-made PCR results performed on carbapenem-resistant Gram-negative bacteria isolated on rectal swab by selective culture.

Overall carbapenem-resistant strains on selective culture (N 125)	NG-test Carba5	Home-made PCR
*Enterobacterales* (n 104)		
Negative, n (%)	26 (25%)	6 (6%)
Positive, n (%)	78 (75%)	98 (94%)
* K.pneumoniae* (n 67)		
Negative, n (%)	8 (12%)	5 (8%)
Positive, n (%)	59 (88%)	62 (92%)
* E.coli* (n 37)		
Negative, n (%)	18 (49%)	1 (3%)
Positive, n (%)	19 (51%)	36 (97%)
*A.baumannii* (n 21)		
Negative	21 (100%)	1 (5%)
Positive	0	20 (95%)

Results of home-made PCR targeting *bla*_KPC_-, *bla*_VIM_-, *bla*_IMP_-, *bla*_OXA_-, *bla*_NDM_- type genes on the 125 carbapenem-resistant strains detected by culture did not find any resistant-gene encoding for carbapenemase in 7 strains (5 K*. pneumoniae*, 1 *E.coli* and 1 A*. baumannii*) and at least a resistant-gene encoding for carbapenemase in 98 (94%) of carbapenem-resistant *Enterobacterales* and in 20 (95%) of carbapenem-resistant *A.baumannii* (**Table 5**).

Carba5 test found KPC in 57 carbapenem-resistant strains (73%), followed by VIM in 23% (18 strains). Only two strains tested positive for NDM and one for OXA-48-like carbapeneamase (**Table 6**).

**Table 6:** Carbapenemases identified in carbapenem-resistant Gram-negative bacteria isolated on selective culture by NG Carba5 rapid test and genes encoding for carbapenemases identified in carbapenem-resistant Gram-negative bacteria isolated on selective culture by home-made PCR.

Variables	Carbapenem-resistant *K.pneumoniae* (N 67)	Carbapenem-resistant *E.coli* (N 37)	Carbapenem-resistant *A.baumanni* (N 21)	Total number of carbapenem-resistant strains (N 125)
Positivity by NG Carba5 rapid test				
Number of strains positive for at least one carbapenemase, n (%)	59 (88%)	19 (51%)	0 (0%)	78 (62%)
Type of expressed carbapenemase KPC, n (%)	48 (81%)	9 (47.5%)	0	57 (73%)
VIM, n (%)	9 (15%)	9 (47.5%)	0	18 (23%)
NDM, n (%)	1 (2%)	1 (5%)	0	2 (2.5%)
OXA-48like, n (%)	1 (2%)	0	0	1 (1.5%)
OXA-23like, n (%)	0	0	0	0
OXA-24like, n (%)	0	0	0	0
Total number of expressed carbapenemases	59 (100%)	19 (100%)	0	78 (100%)
Positivity by home-made PCR				
Number of strains positive for at least one gene encoding for carbapenemase, n (%)	62 (92.5%)	36 (97%)	20 (95%)	118 (94.4%)
bla_KPC,_ n (%)	51^*#^ (82%)	24^‡ ±^ (66%)	0	75 (64%)
bla_VIM,_ n (%)	11^#†^ (18%)	14^‡^ (39%)	0	25 (21%)
bla_NDM,_ n (%)	4^*†^ (6%)	2^±^ (6%)	0	6 (5%)
bla-_OXA48like,_ n (%)	1^†^ (2%)	0	0	1 (1%)
bla-_OXA23like,_ n (%)	0	0	18^°^ (90%)	18 (15%)
bla-_OXA24like,_ n (%)	0	0	20^°^ (100%)	20 (17%)
Total number of identified genes encoding for carbapenemases^•^	67 (108%)^•^	40 (111%)^•^	38 (190%)^•^	145 (123%)^•^

*Two strains harbouring at the same time bla_KPC_ and bla_NDM_; #one strain harbouring at the same time bla_KPC_ and bla_VIM_; † one strain harbouring at the same time bla_OXA-48like_, bla_VIM_ and bla_NDM_; ‡ 3 strains harbouring at the same time bla_KPC_ and bla_VIM_; ± 1 strain harbouring at the same time bla_KPC_ and bla_NDM_; ° 18 strains harbouring at the same time bla_OXA-23_ and bla_OXA-24_; • more than 100% because some strains harbouring at the same time more than one gene for carbapenemase.

Home-made PCR identified *bla*_KPC_ in 75 strains (64%), *bla*_VIM_ in 25 (21%), *bla*_NDM_ in 6 strains (5%) *bla*_OXA-23-like_ in 18 strains (15%), *bla*_OXA-24-like_ in 20 strains (17%), and *bla*_OXA-48-like_ in only one strain (**Table 6**). Both *bla*_KPC_ and *bla*_VIM_ were found in 4 strains and both *bla*_KPC_ and *bla*_NDM_ in 3 strains. Finally, one *K. pneumoniae* strain tested positive for *bla*_OXA-48-like_ /*bla*_NDM_/*bla*_VIM_ simultaneously (**Table 6**).

### 3.3 Enteric colonization by *C. difficile*

Overall, 1147 (59%) of the enrolled residents provided a sample, 134 (11.7%) of which tested positive for GDH. Real time PCR tested positive for toxins A-B genes in 21 GDH-positive strains (15.7%).

### 3.4 Risk factors for colonization by MDR Gram negative organisms and *C. difficile*

The presence of at least one device resulted significantly associated with all outcomes: enteric colonization by III-generation cephalosporin-resistant Gram-negative bacteria, enteric colonization by carbapenem-resistant Gram-negative bacteria end enteric colonization by *C.difficile* (**Table 7**). Antibiotic treatment in the previous 3 months was significantly associated only with colonization by III-generation cephalosporin resistant Gram-negative bacteria. Hospitalizations in the previous year was significantly associated only with colonization by carbapenem-resistant Gram-negative bacteria.

**Table 7:** Risk factors for enteric colonization by III-generation resistant Gram-negative bacteria, carbapenem-resistant Gram-negative bacteria and by *C.difficile*.

OUTCOME	OR*	95%CI	p-value	
Colonization by III-generation cephalosporin – resistant Gram-negative bacteria				
*Presence of at least one device*	*2.67*	*2.06-3.47*	*0.000*	
*Antibiotic treatment in previous 3 months*	*1.48*	*1.19-1.83*	*0.000*	
Hospitalizations in previous 12 months	1.31	0.99-1.72	0.058	
Colonization by carbapenem-resistant Gram-negative bacteria				
*Presence of at least one device*	*2.67*	*1.71-4.17*	*0.000*	
Antibiotic treatment in previous 3 months	1.41	0.91-2.19	0.12	
*Hospitalizations in previous 12 months*	*1.80*	*1.10-2.92*	*0.019*	
Colonization by *C.difficile*				
*Presence of at least one device*	*2.30*	*1.49-3.57*	*0.000*	
Antibiotic treatment in previous 3 months	1.11	0.73-1.68	0.62	
Hospitalizations in previous 12 months	1.19	0.72-1.97	0.50	

*Obtained by two-level random-intercept logistic regression models, with gender and age as adjustment variables.

## 4 Discussion

The LTCFs participating in this study have similar characteristics to those described by HALT2 and HALT3, both in terms of type of facility (general/mixed nursing homes), and of occupancy rate and care organization (European Centre for Disease Prevention and Control, 2014; Ricchizzi et al., 2018). The population described also has characteristics comparable to those of other PPSs (Arvand et al., 2013; McKinnell et al., 2019; Broussier et al., 2020; McKinnell et al., 2020; Kohler et al., 2022), being composed predominantly of females with an average age close to 85 years, and with a length of stay in the structure exceeding one year. Furthermore, the subjects housed in LTCFs prove to be clinically complex, with more than two thirds affected by concomitant chronic pathologies and presenting a high degree of disability, documented by both cognitive and motor impairment. The prevalence of fecal and urinary incontinence above 50% is also similar to that found in other studies (European Centre for Disease Prevention and Control, 2014; Ricchizzi et al., 2018; Broussier et al., 2020). Compared to Boussier et al., on the other hand, a greater frequency of bed rest (25% vs 9%) and a more frequent use of devices (20% vs 6%) was reported, although urinary catheter remains the most used device. Hospitalization in the previous year involves 14% of subjects, in accordance with Arvand et al, but lower than 23%, as reported by Boussier. Exposure to antibiotic therapy in the previous 3 months is significant (about 30% of subjects), lower than that reported in France (41%) but decidedly higher than Austria (16%) (Arvand et al., 2013; Broussier et al., 2020). In our survey, moreover, the use of fluoroquinolones and III-generation cephalosporins is higher, 32% vs 4% and 22% vs 12% respectively. The characteristics described in the examined population confirm not only the complexity and fragility of the elderly subjects resident in LTCFs but also define a population which is constitutively at risk of colonization by MDR germs, being advanced age, the presence of medical devices, the presence of mental and mobility impairment, previous hospitalization and previous antibiotic use, known risk factors for colonized status (Rodríguez-Villodres et al., 2021). Of note in our study the antibiotics mainly prescribed in the previous three months are fluoroquinolones, III-generation cephalosporins and penicillins, antibiotic classes of extensive use within LTCFs due to their broad spectrum of activity and their handling, but also known as independent risk factors for enteric colonization by multi-antibiotic resistant microorganisms (Rodríguez-Villodres et al., 2021).

In this multicenter PPS, a remarkably high rate of colonization with MDR-GNB is observed among LTCF residents in the Veneto region. Just over half (51%) of included residents are colonized with GNB resistant to III generation cephalosporins and 6% with GNB resistant to carbapenems.

In comparison with previous investigations, the colonization rate by III-generation resistant GNB is strikingly higher than that found in Japanese (Nakai et al., 2022) and in other European countries (Jonsson et al., 2011; Arvand et al., 2013; Kohler et al., 2022), although our findings are in line with other Italian reports (Aschbacher et al., 2016; Aschbacher et al., 2020). In the present study, this high rate is mostly due to *E.coli* (65%), suggesting that LTCFs might be a reservoir of this microorganism for the healthcare system.

As already described elsewhere (Cherubini et al., 2022), most of the *E. coli* strains isolated from our population belong to ST131 lineage, mainly of the O25b:H4 serotype and H30 subclone, and ESBL production is mainly attributed to enzymes of the CTX-M -1 group (CTX-M-1, CTX-M-3 and CTX-M-15). In the analysed strains, however, other resistance genes were also found, ST131 representing an incubator of fluoroquinolones, and other antibiotic resistance genes (Cherubini et al., 2022). These results are in line with previous studies conducted in Italian and European acute-care hospitals, indicating that the CTX-M types have disseminated in *E.coli* species. This reflects the selective pressure exerted by frequent and repeated antibiotic therapies especially with fluoroquinolones and III-generation cephalosporins, as already widely described (Urbánek et al., 2007; Fuzi et al., 2020) and documented also by our case series. Furthermore, the high colonization rate can be attributed to the increased probability of horizontal transmission favoured by the care needs of this population, especially for personal hygiene, and by LTCFs’ setting itself, generally characterized by understaffing and sub-optimal hydro alcoholic’s consumption for hand-hygiene (European Centre for Disease Prevention and Control, 2014; Ricchizzi et al., 2018). In line with that described in other surveys, also in our case the recent consumption of antibiotics was found to be risk factors for colonization by Gram negatives resistant to III-generation cephalosporins (Aschbacher et al., 2016; Aschbacher et al., 2020; Broussier et al., 2020).

As recently described, the prevalence of CR GNB colonization within LTCFs is extremely heterogeneous, ranging from 6% to 30% in the USA, 0% to 23% in Asia and reaching 12% in Israel (Chen et al., 2021). In Europe, the reported frequencies range from 0% in Belgium and the Netherlands to 0.3% in Switzerland; in Italy a prevalence of carbapenemase-producing *Enterobacterales* of 6.3% in 2008 and of 1% in 2015 has been reported (Aschbacher et al., 2016). Our population documents a CR GNB colonization rate of 6%, mainly due to carbapenemase encoding GNB as documented by PCR results. These data reflect the frequency of carbapenem-resistant strains also described in the latest EARSS report and related to invasive infections (European Centre for Disease Prevention and Control, 2022). The high frequency of carbapenemase-producing *Enterobacterales*, especially *K.pneumoniae*, is also in line with the EuSCAPE study, according to which the carbapenemase-producing *Enterobacterales* (CPE) colonization/infection rate is higher in the Mediterranean region with a particularly high incidence in Italy (5.96/10,000 hospital admissions) (Grundmann et al., 2017), and are also in accordance with recently described results from Spain (Callejón Fernández et al., 2022). Therefore, our finding of recent hospitalization as a risk factor for colonization by CR GNB is not surprising, although we do not have local epidemiological data on the frequency of colonization/infection by CR GNB in patients admitted between June 2018 and June 2019 in the reference acute care hospitals for the LTCFs included in the present study. With this limit it is not possible to exactly define the circulation of CR GNB between LTCFs and acute care hospitals within the Veneto region. In our study, recent antibiotic use is not associated with colonization by carbapenem-resistant germs. Since the colonization by CR GNB detected through selective culture and immuno-enzymatic test has been considered as an outcome variable, this lack of association could be the consequence of the underestimation of patients colonized by carbapenemase-producing *Enterobacterales* using this method if compared to home-made PCR results. In accordance with other series, *K. pneumoniae* was the most frequently isolated carbapenem-resistant micro-organism (Chen et al., 2021). As already described elsewhere (Piccirilli et al., 2021), in this study the most widespread clones were represented by ST307 and ST512 and the more detected carbapenemases were the KPC variants (KPC-2, KPC-3, and KPC-9), followed by VIM-1. This reflects the Italian epidemiology, both with respect to acute care hospitals and to LTCFs (Aschbacher et al., 2020), and documents how LTCFs represent an important incubator not only of ESBLs but also of carbapenemases.

Finally, the colonization by *C. difficile* is higher than that documented by Giufrè et al. (11.7% vs 5.1%) (Giufrè et al., 2017), despite the lower sensitivity of GDH qualitative enzyme immunoassay adopted as screening test in asymptomatic subjects compared with selective culture used by the other group. Only 2% of *C.difficile* strains was toxigenic, in line with that described by Leitner (Leitner et al., 2018), but clearly lower than, above all, US, and Canadian epidemiology (Donskey et al., 2018; Mallia et al., 2018). This could be linked both to the low sensitivity of the adopted screening test and to the small sample size, since it was possible to collect the samples of only half of the enrolled residents.

This study has several limitations. The point-prevalence design did not consider the dynamics of ESBL and carbapenemasescarbapenems epidemiology, but it was chosen because of its feasibility when applied in settings with limited resources for antimicrobial resistance surveillance, as is the case for LTCFs. We also did not have local epidemiological data to assess the frequency of infection/colonization by III-generation cephalosporin- and carbapenem-resistant *Enterobacterales* and non-lactose fermenters bacteria in individuals admitted in the same period (2018–2019) in the acute-care hospitals of reference for the LTCFs involved in the present study. Furthermore, the screening of healthcare workers and staff was not performed, therefore their role in the MDR-GNB transmission can be only hypothesised but cannot be assessed.

The strengths of this survey include the use of a standardized protocol across all participating LTCFs, the collection of detailed data on the LTCF characteristics and the inclusion of a wide variety of LTCF residents and data on their antimicrobial use. The survey is characterized by a broad participation and a very large sample size, providing a good overall picture of antimicrobial use and MDR GNB colonization’s rate. Finally, we performed Whole Genomic Sequencing on a subset of *E.coli* and *K.pneumoniae* strains isolated by selective culture (data shown elsewhere) (Piccirilli et al., 2021; Cherubini et al., 2022).

## 5 Conclusions

LTCFs are important reservoirs for MDR organisms, mainly III-generation cephalosporins-resistant *Enterobacterales* but also carbapenem-resistant GNB, which can be selected as a result of the antibiotic pressure exerted by repeated antibiotic treatments and/or derive from horizontal transmission mechanisms. Both events may occur within the LTCF or within the acute care hospital on previous admissions.

To contrast the horizontal spread of these microorganisms, the identification and screening of residents coming from nursing homes at hospital admission must be considered (Chen et al., 2021). According to LTCF infection prevention and control guidelines, active surveillance of MDROs in LTCFs by culture methods or by using molecular amplification assays should not imply routine screening of residents at the time of admission to the facility, nor should it be repeated on a periodic basis, in the absence of an epidemic of infections by MDROs, because the application of standard precautions, as applied to all residents, is sufficient (Smith et al., 2008). Carbapenemase-producing *Enterobacterales*, especially KPC-producing *K. pneumoniae*, are epidemically spread in Italy, and infection control and prevention measures in LTCFs is an effective strategy to reduce carbapenem-resistant *Enterobacterales* acquisition and transmission in high endemicity epidemiological setting, such as this one (Toth et al., 2017). The ESCMID-EUCIC clinical guidelines do not recommend routine decolonization of these microorganisms (Tacconelli et al., 2019) because of the potential of increased antimicrobial resistance to decolonizing agents (Tacconelli et al., 2019). Rigorous application of contact precautions has been effective in controlling carbapenem-resistant *Enterobacterales* (Mody et al., 2013), however they are not practical for most LTCFs’ residents due to concerns for unique harms in this setting, which is perceived and experienced as a home (Mody et al., 2013). Focusing on other type of effective measures, such as the importance of better environmental cleaning protocols, more rigorous application of routine infection prevention protocols, and adherence to hand hygiene indications, is of extreme importance.

Antimicrobial use in LTCFs is a critical issue as well (Ricchizzi et al., 2018). Antimicrobial stewardship programs are needed to control the selection of multidrug resistant organisms, supporting physicians not only in the implementation of updated antibiotic treatment guidelines for the most frequent infective syndromes (e.g. upper/lower respiratory tract infections, urinary tract infections, skin and soft tissue infections), but also in the adoption, for example, of point of care diagnostic tests and/or diagnostic algorithms to reduce unnecessary use of antibiotics in LTCFs (Oliva et al., 2018).

## Data availability statement

The original contributions presented in the study are included in the article/supplementary materials, further inquiries can be directed to the corresponding author/s.

## Ethics statement

The studies involving human participants were reviewed and approved by Ethics Committee for Clinical Trials of the Provinces of Verona and Rovigo -Approval n° 25294, 26/04/2018. The patients/participants provided their written informed consent to participate in this study.

## Author contributions

AMA conceived the study, organized the collection of biological samples and clinical data, supervised the statistical analysis, and drafted the manuscript. GB participated in the collection of biological samples and clinical data, participated in the statistical analysis, and drafted the manuscript. GLC, LN and AB performed the microbiological analysis. JM and MM conducted the statistical analysis. IC, NDS and LL collected biological samples and clinical data, all of them also participated in data entry. FM helped in the coordination of sample and clinical data collection. ET and GLC conceived the study. All authors reviewed and approved the final manuscript. All authors contributed to the article and approved the submitted version.
